# Adolescents’ short-form video addiction and sleep quality: the mediating role of social anxiety

**DOI:** 10.1186/s40359-024-01865-9

**Published:** 2024-06-28

**Authors:** Li Jiang, Yizoon Yoo

**Affiliations:** 1 Physical Education Institute, Yantai University, Yantai, China; 2 Chonnam University, Jeollanam do, Gwangju, South Korea

**Keywords:** Short-form video addiction, Sleep quality, Social anxiety, Adolescents

## Abstract

**Background:**

Adolescence is a critical period for individual growth and development. Insufficient sleep adversely affects adolescents’ physical development, blood pressure, vision, and cognitive function. This study examined the effect of short-form video addiction on adolescents’ sleep quality, as well as the mediating role of social anxiety, to identify methods for improving adolescents’ sleep quality in the Internet era.

**Methods:**

A questionnaire survey was conducted in this cross-sectional study on 1629 adolescents recruited from three high schools. Their short-form video addiction, social anxiety, and sleep quality were evaluated using corresponding scales. Pearson correlation analysis was carried out to analyze the relationships among short‐form video addiction, sleep quality, and social anxiety. Mediating effect analysis was constructed using AMOS 20.0 statistical software.

**Results:**

Participants’ sleep quality score is 6.12 ± 3.29 points. The detection rate of sleep quality among them is 31.06%. Short‐form video addiction, sleep quality, and social anxiety are significantly correlated (*r* = 0.439, 0.404, 0.457, *P* < 0.001). The direct effect of short-form video addiction on sleep quality is 0.248, accounting for 62.4% of the total effect. The indirect effect exerted through social anxiety is 0.149, accounting for 37.6%.

**Conclusions:**

Sleep disorders are very common among Chinese adolescents. Short‐form video addiction is positively correlated with adolescents’ sleep quality and social anxiety. Social anxiety partially mediates the relationship between short-form video addiction and sleep quality. The adverse effects of short-form video addiction and social anxiety on the sleep quality of this group must be minimized. Schools are recommended to implement measures to promote sleep quality among adolescents.

## Introduction

Sleep is one of the most basic physiological requirements for human beings. Adequate sleep not only promotes bodily growth, physical development, and immune function regulation but also contributes significantly to mental health [1]. Adolescents are in a special period of physical, cognitive, emotional, and interpersonal changes, during which sleep has unique implications for their growth [2]. Compared with college students and children, adolescents have more prominent sleep problems, with a higher incidence of sleep disorder and a trend of increasing deterioration [3]. Besides short sleep hours, adolescents have serious problems with sleep quality [3]. Sleep problems lead to a series of adverse outcomes, including decreased immune function, obesity, traffic accidents, substance abuse, depression, and suicidal tendency [1–3]. Psychological factors such as resilience, loneliness, depression, and stress can decrease individuals’ sleep quality [4]. Physiological and behavioral factors such as fatigue and excessive use of the Internet and smartphones can also affect adolescents’ sleep quality [5].

Short-form video, a new type of Internet-based content dissemination method emerging in recent years, has gradually become an important factor affecting adolescents’ sleep quality. Short-form videos are highly popular because of their rich and diversified content, favorable human–computer interaction mode, easy access, and short viewing time [6]. The number of short-form video users in China had reached 934 million by December 2021 [7]. As pointed out in “Blue Book of Teenagers: Annual Report on the Internet Use by Minors in China (2021),” adolescents have become the largest group of short-form video users [7]. A study on the negative effects of Internet use reveals that an increase in online time can lead to emotional changes, depression, and intense psychological conflicts, negatively affecting daily life. As a new form of Internet addiction, short-form video addiction refers to a chronic or periodic obsession state featured by the repeated use of short-form video apps (such as Tiktok, Kwai, etc.), resulting in strong and continuous craving and addiction [8]. Among the youth population in China, 49.3% of underage netizens watch short videos online. In addition, 60.4% of adolescent short video users reported watching short videos on Tiktok, and 59.3% of them reported watching them on Kwai [9]. Ye et al. [10] found in their research on college students that short-form video addiction exacerbates the negative effects on students’ academic and physical and mental health, for example, reducing learning motivation and increasing depression incidence. In short, this addictive behavior causes many negative effects, including psychological problems [10, 11]. Zhang et al. [12] conducted a cross-sectional survey on Internet addiction and sleep quality in Vietnamese adolescents and found a significant positive correlation between Internet addiction and sleep quality. Internet addiction is a high-risk factor for poor sleep quality [12]. Short-form video addiction, as a new form of Internet addiction, exhibits the general characteristics of addictive behavior, such as strong and sustained cravings and psychological and behavioral addiction. Therefore, short-form video addiction may affect adolescents’ sleep quality, and the mechanism through which short-form video addiction affects sleep quality must be investigated.

Social anxiety is a phenomenon individuals experience in multiple social situations caused by fear that their words and actions may be negatively evaluated by others. Severe social anxiety can also cause social anxiety disorders in individuals [13, 14]. A study using the cross-lagged model to examine the relationship between sleep and psychological symptoms finds that high levels of anxiety predict a sustained decrease in sleep time and quality [15]. Another study on young people shows that low levels of positive emotions and high levels of negative emotions are closely related to poor sleep quality [12]. Liu et al. conducted an online questionnaire survey on 1402 middle school students in China and found that social anxiety significantly predicts poor sleep quality [16]. People without sufficient sleep experience a decrease in their perception of happy emotions [16]. This biased emotional perception is associated with social dysfunction and psychological problems, and social anxiety is a common psychological problem among adolescents. Therefore, social anxiety, as a type of anxiety [17], negatively affects adolescents’ sleep quality.

A significant positive correlation is also observed between Internet addiction and social anxiety. Li et al. [18] suggested that as Internet addiction increases, adolescents spend more time and energy on the Internet and less on activities such as interpersonal communication. This imbalance results in decreased self-efficacy and increased loneliness, ultimately leading to social anxiety. According to the “uses and gratifications approach,” short-form video and other Internet social media provide adolescents who are unable to meet their belonging needs or maintain social relationships in real life with more opportunities to interact with others [18]. However, long-term addiction to virtual social media greatly increases the risk of individuals relying on short-form video. Adolescence is a critical period for the development of social anxiety [19]. Currently, studies on the relationship between short-form video addiction and social anxiety in this group are few. Hence, examining the internal relationship between short-form video addiction and social anxiety is necessary.

In summary, short-form video addiction may affect adolescents’ sleep quality, social anxiety may also affect their sleep quality, and short-form video addiction may significantly predict social anxiety. Focusing on adolescents, this study explores the mechanism through which short-form video addiction affect their sleep quality. The goal is to provide valuable reference for preventing short-form video addiction and improving sleep quality among adolescents.

## Research methods

### Research participants

With reference to previous experience, large-scale sample surveys aim to make the selected samples representative. According to Kline [20], the sample size must be more than 20 times the number of items. Therefore, the sample size should be at least 860, considering the 43 items.

Using the stratified cluster sampling method, a questionnaire survey was conducted between June and July 2023 on first-year and second-year high school adolescents in Shandong Province, China. Third-year high school students were excluded in the scope of this survey because of their need to spend more time preparing for the upcoming college entrance examination in China. After obtaining informed consent from each participant, copies of the questionnaire were distributed to each class. The survey was conducted in the form of collectively filling out the printed questionnaire. Members of the research team explained the filling requirement before they asked participants to fill out the questionnaire. They were required to complete the questionnaire within the specified time. Those who met the following criteria were included: those with Chinese nationality; those proficient in Chinese with basic listening, speaking, reading, and writing skills; first- and second-year high school students; those without other confirmed mental illness; and those who have not participated in similar research before. Exclusion criteria were those who did not meet the inclusion criteria and those with reading disorders.

A total of 1655 copies of the questionnaire were distributed, and 1650 copies were collected. After checking the copies of the questionnaire collected, 21 copies were found to be filled out regularly, with all items unanswered or with some items unanswered. These copies were considered invalid. After 21 copies with regular or missing filling were excluded, 1629 valid copies remained, with an effective recovery rate of 98.4%. Among them, 832 and 797 copies were collected from first-year and second-year high school students, respectively, with an average age of 16.54 + 0.98 years old. The questionnaires were filled out by 831 male students (51.0%) and 798 female students (49.0%). Table 1 provides additional demographic information. Before conducting the survey, members of the research team explained the significance, methods, and precautions of the survey to all the participants, guardians, and school administrators. They emphasizes that the survey was conducted anonymously and that all data are for scientific research purposes only. The personal information of the participants would be strictly protected. Informed consent to participate was obtained from all of the participants in the study. This survey meets the requirements of the Ethics Committee.

### Measurement instruments

#### Short-form video addiction scale [21]

This study referred to the Short Video-dominated Social Media Dependence Scale adapted by Hu et al. [21]. The scale was revised from the Social Network Dependence Scale prepared by Milošević-Đorđević [22]. “Social networking sites” in each item was changed to “short video social networking sites”. The scale consists of 6 items, scored from 1 indicating “highly disagree” to 5 indicating “highly agree” on a 5-point scale. A higher score means a higher degree of short-form video addiction. The internal consistency of this scale for measuring short-form video addiction among Chinese adolescents is 0.732 [21].

#### Social interaction anxiety scale (SIAS) [23]

SIAS was prepared by Mattick and Clarke [24] and revised by Chinese scholars Ye et al. [23]. SIAS measures anxiety and fear of expressing and being observed in social situations based on the description of social phobia in DSM-III-R. It contains 19 items, with the items 8 and 10 being reverse-scored. Items are scored from 1 (completely disagree) to 5 (completely agree) on a 5-point scale. A higher score indicates a higher level of social anxiety. When SIAS is used with Chinese adolescents, the Cronbach’s α coefficient is 0.874 [23].

#### Pittsburgh sleep quality index (PSQI) [25]

PSQI is a self-rating sleep quality scale compiled by Buysse et al. [26] and then translated into Chinese by Liu et al. [25] for application in China. PSQI consists of 18 items, including 3 fill-in-the-blank items, 5 multiple-choice items, and 10 self-rating items. The total score on this scale is the sum of scores in seven factors, namely, sleep quality, sleep latency, sleep hours, sleep efficiency, sleep disorder, hypnotics, and daytime dysfunction. The total score on this scale ranges from 0 to 21 points. A higher score means poorer sleep quality. A total score greater than 7 on this scale is considered indicative of a sleep disorder. The Cronbach’s α coefficient of PSQI is 0.77 when applied to the Chinese population.

### Statistical analysis

The data were analyzed using SPSS 20.0 statistical software, and the quantitative data that followed normal distribution were expressed as mean ± standard deviation. Independent sample t-test was used for comparison between two groups; one-way ANOVA was used for comparison among multiple groups. Student–Newman–Keuls method was used for pairwise comparison; Pearson correlation analysis was used for analyzing the correlation between two variables. AMOS20.0 statistical software was used for analysis of mediating effect, and the maximum likelihood method was used for parameter estimation. A difference was considered statistically significant if *P* < 0.05.

### Quality control

The following measures were taken to reduce response bias or social desirability effects in the self-report measures: (1) Prior to data collection, all members of the research team participated in collective training. The training included selecting participants strictly according to the inclusion criteria; specifying the wordings used for offering questionnaire filling guidance and explanation; discussing the problems and solutions possibly encountered during questionnaire collection; and standardizing the steps of questionnaire distribution, filling out, and collection. (2) During data collection, participants were informed that this study would be conducted anonymously without privacy disclosure to obtain their cooperation. After questionnaire completion, the researchers checked all items individually to ensure data integrity. (3) After summarizing the collected data, two members verified and input them. (4) While the participants were filling out the questionnaires, the researchers did not provide any suggestions or hints related to the study results.

## Results

### Analysis of status and differences in short-form video addiction, social anxiety, and sleep quality

Adolescents have a short-form video addiction score of 13.79 ± 4.36, social anxiety score of 42.76 ± 14.23, and sleep quality score of 6.12 ± 3.29 (detection rate = 31.06%). No statistically significant differences are found in the short‐form video addiction score based on grade, only child status, gender, and family residence (*P* > 0.05). However, statistically significant differences are observed based on academic performance, family economic condition, and exercise frequency (*P* < 0.05). No statistically significant differences are found in the social anxiety score based on grade, only child status, gender, family residence, and exercise frequency (*P* > 0.05). Statistically significant differences are observed though based on academic performance and family economic condition (*P* < 0.05). No statistically significant differences are found (*P* > 0.05) in the sleep quality score based on grade, only child status, and family residence. Yet, statistically significant differences are noted (*P* < 0.05) based on gender, academic performance, family economic condition, and exercise frequency. The results are shown in Table 1.

**Table 1:** Differences in short-form video addiction, social anxiety, and sleep quality by different demographic factors

Demographic factor		*n*	short-form video addiction	Social anxiety	Sleep quality
Grade	First year	832	13.78 + 4.23	43.15 + 14.73	6.22 + 3.36
	Second year	797	13.79 + 4.49	42.36 + 13.69	6.03 + 3.21
	*t*		0.043	1.127	1.216
	*P*		0.966	0.260	0.224
Whether the only child	Yes	671	13.64 + 4.36	42.24 + 14.05	6.01 + 3.25
	No	958	13.89 + 4.36	43.13 + 14.35	6.21 + 3.32
	*t*		1.157	1.250	1.188
	*P*		0.247	0.211	0.235
Gender	Male	831	13.86 + 4.34	42.30 + 13.89	5.94 + 3.20
	Female	798	13.71 + 4.38	43.24 + 14.58	6.32 + 3.37
	*t*		0.728	1.329	2.338
	*P*		0.467	0.184	0.020
Family residence	Rural areas	837	13.97 + 4.45	43.38 + 14.45	6.11 + 3.29
	Urban areas	792	13.59 + 4.26	42.11 + 13.98	6.14 + 3.29
	*t*		1.744	1.794	0.194
	*P*		0.081	0.073	0.846
Academic performance	Poor (A)	158	15.07 + 4.73	41.57 + 12.14	5.90 + 2.96
	Slightly poor (B)	291	14.73 + 4.56	44.00 + 15.73	6.57 + 3.37^a^
	Medium (C)	591	13.70 + 4.34^ab^	44.17 + 14.78^a^	6.25 + 3.50
	Slightly good (D)	430	13.20 + 4.13^a^	40.88 + 13.17^bc^	5.84 + 3.13^bc^
	Good (E)	159	12.69 + 3.66^abc^	41.56 + 13.28	5.86 + 2.98^b^
	*F*		11.608	4.475	2.846
	*P*		< 0.001	0.001	0.023
Economic condition	Poor (A)	296	11.50 + 4.04	41.01 + 14.72	5.96 + 3.37
	Slightly poor (B)	337	12.54 + 3.60^a^	39.64 + 13.12	5.15 + 3.36^a^
	Medium (C)	573	14.51 + 4.25^**ab**^	43.93 + 14.32^**ab**^	6.39 + 3.30^**b**^
	Slightly rich (D)	274	15.05 + 4.60^ab^	43.51 + 14.01^**ab**^	6.54 + 2.89^a**b**^
	Rich (E)	149	16.02 + 3.78^abcd^	47.45 + 13.94^**abcd**^	6.89 + 3.18^a**b**^
	*F*		11.608	4.475	2.846
	*P*		< 0.001	0.001	0.023
Exercise frequency	Nearly no (A)	115	14.65 + 5.06	43.23 + 15.29	6.42 + 3.45
	1–3 days/week (B)	938	14.31 + 4.34	43.12 + 14.15	6.26 + 3.12
	3–5 days/week (C)	446	12.80 + 3.99^ab^	42.33 + 14.11	5.94 + 3.67
	Over 5 days/week (D)	130	12.65 + 4.25^ab^	41.25 + 14.29	5.53 + 2.92^ab^
	*F*	0	17.054	0.862	2.715
	*P*	0	< 0.001	0.460	0.043

*Note*^a^ presents *P* < 0.05 compared to Group A; ^b^ presents *P* < 0.05 compared to Group B; ^c^ presents *P* < 0.05 compared to Group C; ^d^ presents *P* < 0.05 compared to Group D

The Pearson correlation analysis shows pairwise positive correlations among short-form video addiction, social anxiety, and sleep quality. As shown in Table 2, short‐form video addiction is significantly positively correlated with sleep quality (*r* = 0.404, *P* < 0.05), short‐form video addiction is significantly positively correlated with social anxiety (*r* = 0.439, *P* < 0.05), and sleep quality is significantly positively correlated with social anxiety (*r* = 0.457, *P* < 0.05).

**Table 2:** Correlation analysis among short-form video addiction, social anxiety, and sleep quality

Variable	*r*	*P*
short-form video addiction and social anxiety	0.439	< 0.001
short-form video addiction and sleep quality	0.404	< 0.001
sleep quality and social anxiety	0.457	< 0.001

### Analysis of mediating effect

**Fig. 1:**
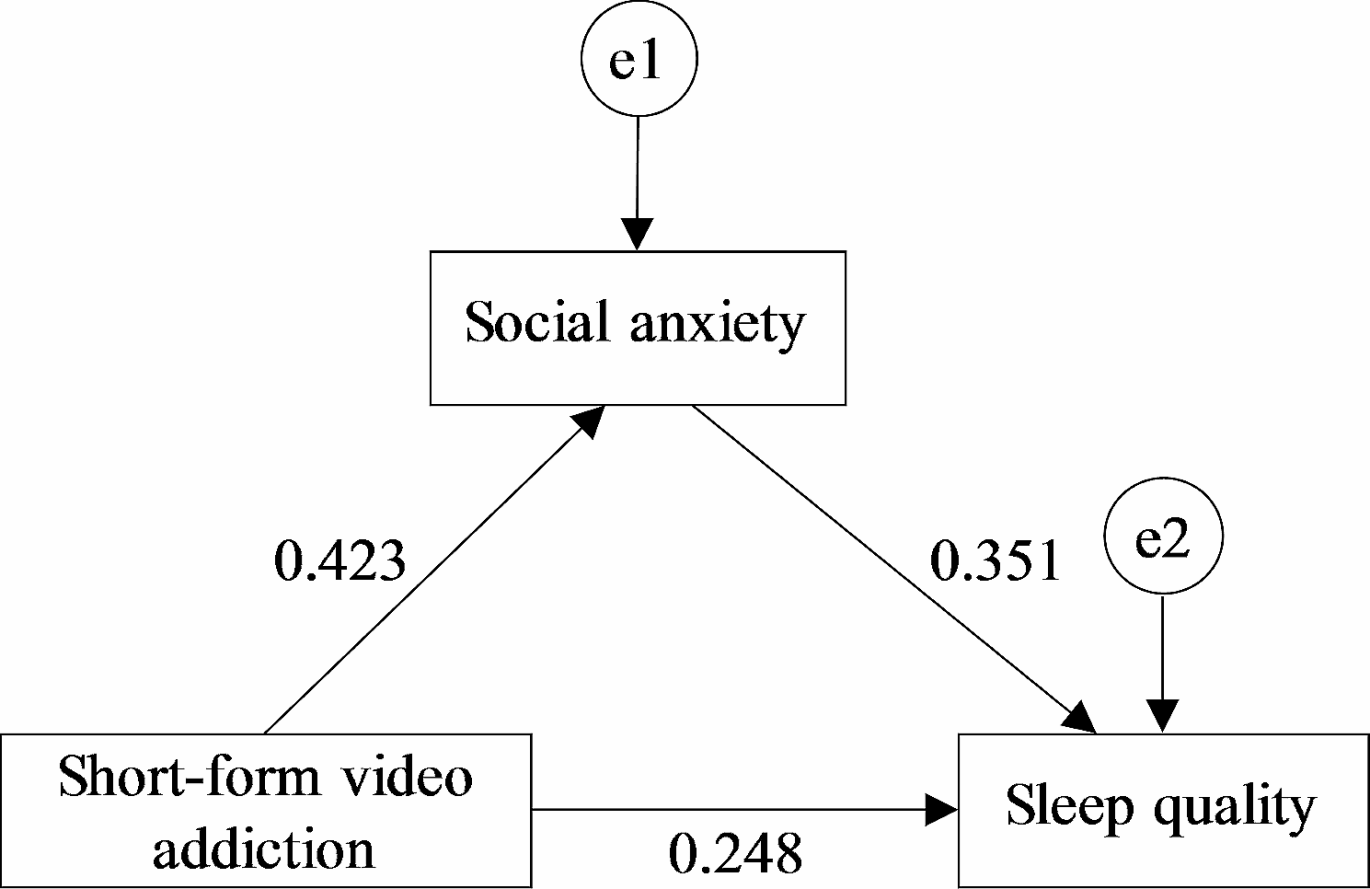
Mediating effect model (standard regression coefficient)

To further explore the relationships among variables and test whether social anxiety mediates the relationship between short-form video addiction and sleep quality, this study established a structural equation model using Amos 21.0. The model had short‐form video addiction as the independent variable, social anxiety as the mediating variable, and sleep quality as the dependent variable. The specific path is shown in Fig. 1. The results of parameter test show that the path coefficients of the three paths are statistically significant (*P* < 0.05), as shown in Table 3.

**Table 3:** Test of path coefficients of the model

Path			Non standardized path coefficient	Standard error	*t*	*P*
Social anxiety	<---	short-form video addiction	1.376	0.073	18.82	< 0.001
sleep quality	<---	short-form video addiction	0.187	0.018	10.542	< 0.001
sleep quality	<---	Social anxiety	0.081	0.005	14.908	< 0.001

To further examine the direct and indirect effects of short-form video addiction on sleep quality, this study used the bootstrap self-sampling method to calculate the effect values and 95% confidence intervals. The results are shown in Table 4. The direct effect of short‐form video addiction on sleep quality is 0.248 (95% CI: 0.199–0.296), accounting for 62.4% of the total effect value (0.397). The indirect effect exerted through social anxiety is 0.149 (95% CI: 0.120–0.183), accounting for 37.6%. These results suggest that social anxiety partially mediates the effect of short‐form video addiction on sleep quality.

**Table 4:** Analysis of mediating effect values of the model

Effect type	Effect estimate	bootstrap 95% CI		*P*
		Lower limit	Upper limit	
Direct effect	0.248	0.199	0.296	0.019
Indirect effect	0.149	0.120	0.183	0.004
Total effect	0.397	0.366	0.435	0.006

## Discussions

### Status and characteristics of adolescents’ sleep quality

In this study, the total sleep quality score is 6.12 ± 3.29, and the detection rate of sleep disorder is 31.06%. These results are similar to the finding from other studies [27, 28]. Adolescents’ sleep problems still require attention from the education department. Further analysis reveals no significant differences in sleep quality among adolescents based on grade, only child status, and family residence (*P* > 0.05). The similarity in the learning content and environment for first-year and second-year high school students likely explains the lack of significant difference in sleep quality by grade. No significant differences are found in the sleep quality between adolescents who are the only child and who are not and between those who live in rural areas and those who live in urban areas. However, some studies report that only children experience higher levels of emotional warmth and understanding from their parents than non-only children. Their families generally adopt a warm and sympathetic parenting style to establish an affectionate and trusting atmosphere, so only children have higher sleep quality than non-only children [29]. This finding may be related to the emphasis recently placed by school administrators and the educational community on strengthening mental health education among high school students. For example, family-school cooperative management programs such as “Family–School Alliance” help cultivate a sense of security and trust among adolescents, stabilize their emotions, and ultimately improve their sleep quality [30]. This reason also account for the significant difference in sleep quality among adolescents with different family residences.

By contrast, significant differences are found in sleep quality among adolescents based on gender, academic performance, family economic condition, and exercise frequency (*P* < 0.05). Further analysis indicates that girls have poorer sleep quality than boys. This result is probably due to physiological and personality factors that make girls more prone to tension and anxiety. In higher grades, academic performance significantly affects adolescents’ development of mental health [28, 31]. Poor academic performance and greater learning difficulty lead to increased learning pressure, so high-school students experiencing both are prone to negative emotions such as learning anxiety. In addition, the subjective and objective factors such as self-isolation lead to poorer academic performance and sleep quality among adolescents. In addition, this study observes certain relationships between family economic condition and exercise frequency and adolescents’ sleep quality, which is consistent with previous research results [3, 32]. Poorer family economic conditions worsen sleep quality. The reason is the a significant gap in living and educational conditions between those with good family economic conditions and those with poor economic conditions. The latter tends to have increased learning time, perceive pressure and mental tension, and suffer from poorer sleep quality [3]. In this study, 115 high school students have no exercise within a week, and 938 exercise one to three days per week, which to some extent indicates that their physical exercise is worrying. This frequency may be related to high school students having more learning tasks, less free leisure time, and insufficient self-awareness. Strengthening physical exercise is beneficial for reducing the occurrence of anxiety symptoms, and adolescents who exercise more frequently are more likely to have longer sleep hours compared with their peers who exercise less frequently [3, 32]. In the past decade, short-term intervention studies find a significant correlation between objectively measured sleep hours and physical exercise [3, 33, 34].

### Correlation analysis of short-form video addiction, social anxiety, and sleep quality

This study finds that short-form video addiction significantly predicts sleep quality. A higher degree of short‐form video addiction among adolescents results in poorer sleep quality. This result is consistent with the findings from previous studies on Internet addiction among adolescents [4, 5, 31]. During the daytime, high school students may be prohibited by teachers or school administrators from using mobile phones in the classroom because of overloaded academic tasks [35]. Many adolescents may excessively watch short-form video online before going to bed at night, thus decreasing their sleep hours. Besides, the blue light emitted by electronic mobile devices at night can interfere with the secretion of melatonin, a hormone that regulates sleep [36, 37]. The radio frequency electromagnetic fields generated by electronic mobile devices can also disrupt normal blood flow and metabolic functions in the brain, thereby negatively affecting adolescents’ sleep quality [38]. Therefore, this study provides new ideas and references for improving the sleep quality of adolescents.

This study reveals a significant positive correlation between short-form video addiction and social anxiety among adolescents. This outcome is consistent with the research finding that Internet addiction leads to an increase in social anxiety among adolescents [14, 39, 40]. On the one hand, adolescents who rely on short-form video consume plenty of time to watch short-form video. The resulting difficulty in focusing their mind on learning or daily interpersonal communication likely leads to setbacks such as decreased academic performance and hindered interpersonal communication, thus giving rise to social anxiety among adolescents [39]. On the other hand, short-form video platforms feature both high-quality and poor-quality videos, with many videos exhibiting a materialistic bias. Negative content can trigger upward social comparison among adolescents. Long-term upward social comparison on social networking sites likely generates negative emotions, such as depression and jealousy, among adolescents, which are positive correlated with social anxiety [41]. Therefore, negative content further increases the level of social anxiety level coming from short-form video addiction [41]. Research indicates that short‐form video addiction indirectly reduces real-life interactions and contributes to interpersonal relationship barriers [41].

Moreover, social anxiety can increase the use of short videos among adolescents. At present, social anxiety is considered one of the predominant risk factors causing mobile phone addiction [42, 43], and the findings of these studies to some extent support the results of the current study. According to the “uses and gratifications approach” [18], adolescents who struggle to meet their need for belonging or maintain social relationships in real life may turn to short videos for opportunities to interact with others, efficiently satisfying their need for interpersonal communication [18]. Doing so reduces the risk of poor interpersonal relationships and alleviates the pressure arising from unfavorable social environments. As suggested by social cognitive theory, individuals with social anxiety are prone to negative evaluations of their environment and other people. Those with poorer social support systems tend to have more severe social avoidance tendencies, are less able to integrate into the group, or are more likely to be excluded by the group [43, 44]. Thus, they may seek social connections and a sense of belonging by watching short videos, ultimately leading to addiction.

### Mediating role

The mediating effect analysis shows that short-form video addiction has a positive predictive effect on sleep quality (β = 0.248, *P* < 0.05) and that social anxiety partially mediates the relationship between short‐form video addiction and sleep quality among adolescents (β = 0.149, *P* < 0.05). In other words, short‐form video addiction not only directly predicts sleep quality but also indirectly affects sleep quality through social anxiety. The reasons are as follows: (1) Adolescents addicted to short-form videos tend to watch them without supervision from teachers or parents on their mobile phones during night breaks. The electromagnetic radiation generated by mobile phones affects the nervous system, disrupts brain function and metabolism, delays latency, cause dizziness and headaches, and decreases sleep quality [35–37]. (2) Loneliness comes from individuals’ failure to reach their ideal interpersonal communication level. Short‐form video addiction has become an important external factor contributing to loneliness [39]. Excessive Internet use can cause poor adaptation, leading individuals to immerse themselves in the virtual world, which reduces social interactions and creates interpersonal relationship barriers in real life [39, 45]. Ultimately, excessive Internet use exacerbates social anxiety. The rich and diverse content of short-form videos and highly perceivable enjoyment they provide cause adolescents to overuse these videos, resulting in their reduced interpersonal communication and social skills in real life. When facing the real world again, these adolescents feel more detached from society and have a higher level of social anxiety. People with anxiety are likely to have poorer sleep quality and more prominent problems during daytime such as drowsiness, fatigue, and lack of concentration [46]. Individuals with high anxiety also harbor negative attitudes and emotions, exhibiting hostility toward external stimuli. Hostility is significantly correlated with poor sleep quality and ultimately decreases overall sleep quality [33, 46]. Therefore, alleviating adolescents’ social anxiety can help alleviate their short‐form video addiction and improve their sleep quality.

In summary, adolescents’ short-form video addiction has become a social problem that negatively affects their physical and mental health. This study explores the effect of short-form video addiction on the sleep quality of adolescents, and the results indicate the need to actively address sleep quality issues and short‐form video addiction. In addition, alleviating social anxiety can alleviate the negative impact of short‐form video addiction on sleep quality, thereby improving the sleep quality of adolescents and supporting their healthy development.

### Limitations and future direction

This study has some limitations. First, all the data were collected from subjective reports by the participants, which may probably lead to errors, such as memory bias and social desirability bias). Second, the sample was limited to students from three high schools in Shandong Province, China, which may introduce selection bias. Besides, this cross-sectional study lacked follow-up investigation. Future research should collect data from multiple sources (such as individuals, peers, parents, teachers, etc.) to measure relevant variables more objectively. Longitudinal tracking can be conducted to expand the survey scope and verify the internal connections among loneliness, short-form video addiction, and sleep quality as well as the corresponding mechanisms of action.

## Conclusions

The results of this study can provide empirical support and beneficial insights for improving adolescents’ sleep quality, weakening the impact of short-form video addiction in the era of mobile Internet, and maintaining adolescents’ physical and mental health. Sleep quality remains a prominent problem among adolescents that require attention from the education department. Short‐form video addiction has a significant direct effect on adolescents’ sleep quality. Adolescents should learn to consciously suppress their desire to watch short-form video, reduce the frequency of their Internet use, and engage in self-control before falling asleep to ensure sufficient sleep hours. In addition, short‐form video addiction increases social anxiety among adolescents and leads to more negative emotions and less positive emotions, thereby undermining their sleep quality. In response, parents and teachers should consciously guide adolescents to master socializing strategies and improve their interpersonal skills to weaken the mediating effect of negative emotions such as anxiety on the relationship between short‐form video addiction and sleep quality.

## Data Availability

The datasets used and analyzed during the current study are available from the corresponding author on reasonable request.
